# The role of motivation in the association of dispositional mindfulness with self-learning and academic achievement

**DOI:** 10.3389/fpsyg.2025.1541128

**Published:** 2025-06-09

**Authors:** Aamer Aldbyani, Zaid Alhadoor, Zhang Chuanxia, Zhenwen Sheng

**Affiliations:** ^1^Department of General Education, Shandong Xiehe University, Jinan, China; ^2^Faculty of Education, Thamar University, Dhamar, Yemen

**Keywords:** dispositional mindfulness, academic achievement, GPA, intrinsic motivation, extrinsic motivation, self-learning, academic performance

## Abstract

**Introduction:**

Prior research has explored relationships among dispositional mindfulness, motivation, and academic performance, the mechanisms linking these variables remain unclear. This study aims to examine whether dispositional mindfulness predicts self-learning and academic achievement in Yemeni college students, and the potential mediating roles of intrinsic and extrinsic motivation in these associations.

**Method:**

Two hundred and eighty-three Yemeni college students (170 males; 18–26 yrs) completed the Mindful Attention Awareness Scale (MAAS), Personal Learning Scale (PLS), Intrinsic/Extrinsic Motivation Scale, and semester GPAs were obtained from college records.

**Results:**

Dispositional mindfulness correlated moderately with self-learning (*r* = 0.49, *p* < 0.001) and GPA (*r* = 0.43, *p* < 0.001). Intrinsic motivation was associated with dispositional mindfulness (*r* = 0.41), self-learning (*r* = 0.42) and GPA (*r* = 0.50), all *p* < 0.001. PROCESS mediation (Model 4, 5,000 bootstraps) indicated that intrinsic motivation partially mediated the effect of dispositional mindfulness on self-learning (indirect *β* = 0.07, SE = 0.02, 95% CI [0.03, 0.10]) and GPA (indirect *β* = 0.06, SE = 0.02, 95% CI [0.02, 0.09]), accounting for ≈ 28 and 35% of the total effects, respectively. Extrinsic motivation showed small zero-order correlations but did not mediate either outcome.

**Conclusion:**

Higher dispositional mindfulness predicts stronger intrinsic motivation, which in turn fosters sustained self-learning and higher academic achievement; purely extrinsic motives appear insufficient for long-term gains.

## Introduction

The primary goal of educators is to foster academic success, a topic widely discussed in educational literature. As a result, extensive research has examined various factors, such as mindfulness, that may influence or enhance students’ academic performance. Dispositional mindfulness has attracted attention for its benefits in learning. It enables students to develop cognitive and emotional skills that support their educational goals (Alomari, 2023; Bordbar et al., 2024; Kuroda et al., 2022). It is also widely regarded as a foundational construct within the field of positive psychology (Ljubin Golub and Gajšek, 2024; Verhaeghen, 2023).

Numerous studies have explored the relationship between mindfulness and academic performance (Caballero et al., 2019; Güldal and Satan, 2022). Dispositional mindfulness has been found to enhance various academic capacities, including working memory (Derakshan and Eysenck, 2009), recall speed (Bakosh et al., 2016; Brown et al., 2016), and attentional flexibility (Keye and Pidgeon, 2013; Thompson et al., 2011). Furthermore, it positively predicts academic performance, while self-criticism and self-doubt have been identified as negative predictors (Heshmati and Pellerone, 2018). Students with lower levels of dispositional mindfulness tend to perform worse on exams compared to their more mindful peers (Bakosh et al., 2016; Docksai, 2013). For lower-achieving students, a lack of mindfulness may also hinder identity development by impeding the cultivation of reflective and analytical thinking.

Despite growing interest in the links between mindfulness, motivation, and academic outcomes, the underlying mechanisms that connect these variables remain insufficiently understood. Motivation theories, such as self-determination theory, emphasize the importance of intrinsic and extrinsic motivation in fostering student engagement and academic achievement (Gopalan et al., 2017; Park, 1976; Seifert and Sutton, 2018). In parallel, mindfulness theory suggests that motivation may be shaped by present-moment awareness, emotional regulation, and attentional control (Reber, 2014; Tobin, 2018; Ryan et al., 2021). Motivation is increasingly viewed as a key pathway through which mindfulness may influence learning outcomes. According to self-determination theory (van et al., 2012), intrinsic motivation is driven by internal interest and satisfaction, while extrinsic motivation is driven by external rewards. Although both forms are associated with academic success, their specific roles within the context of mindfulness remain underexplored, particularly in non-Western, conflict-affected contexts.

This study seeks to examine whether dispositional mindfulness predicts self-learning and academic achievement among college students in Yemen, a country where prolonged conflict has significantly disrupted the education system. A central contribution of this research is its investigation into the distinct mediating roles of intrinsic and extrinsic motivation in this relationship within a conflict-affected setting.

### Mindfulness and academic performance

In positive psychology, mindfulness is one of the most significant and frequently discussed topics. This field emphasizes the positive aspects of human experience rather than the negative. Mindfulness has been conceptualized as a trait-like tendency, a temporary state of awareness, and a set of skills (Randal et al., 2015). Specifically, this study focuses on dispositional mindfulness, which refers to an individual’s habitual tendency to be attentive to and aware of present-moment experiences in daily life (Brown and Ryan, 2003). Garland and Howard (2013) define mindfulness as paying attention to the present moment openly and objectively. Higher levels of dispositional mindfulness have been linked to greater well-being, empathy, self-acceptance, and compassion for oneself and others (Feltman et al., 2009; Burgoon et al., 2000). Furthermore, dispositional mindfulness has been shown to predict lower levels of stress, depression, psychological distress, and anxiety (Alzahrani et al., 2020). It also supports the development of metacognitive awareness, which involves recognizing thoughts and sensations that require regulation to reduce negative emotions and interrupt maladaptive thinking patterns (Hasker, 2010). A person’s skills, aspirations, motivation, and goal-directed behavior may also influence their mindfulness.

Academic performance refers to an individual’s demonstrated knowledge in a specific subject or area, as well as the extent to which they achieve established educational goals (Steinmayr et al., 2019). Strong academic performance requires goal-directed behaviors, such as acquiring specific skills and mastering relevant content. It is commonly assessed through evaluations in core subjects like mathematics and literacy and reflects students’ cumulative achievements across academic disciplines (McCloskey, 2015). Research suggests that students with higher levels of dispositional mindfulness tend to perform better on exams compared to their less mindful peers (Bakosh et al., 2016; Docksai, 2013). Among high-achieving students, mindfulness may also contribute to identity development by promoting analytical and reflective thinking patterns.

Several studies have examined the relationship between dispositional mindfulness and academic performance. For instance, Caballero et al. (2019) investigated the association between dispositional mindfulness and academic achievement, as well as the reliability of the short-form Mindful Attention Awareness Scale (MAAS) among urban primary school students. Their findings indicated that dispositional mindfulness is positively correlated with academic achievement and that the MAAS is a reliable instrument for measuring mindfulness among youth in academic settings. Similarly, a study by Güldal and Satan (2022) explored the relationships among dispositional mindfulness, character strengths, and academic achievement in high school students. Using the MAAS, the researchers found that mindfulness, along with three-character strengths—persistence, prudence, and openness to learning—accounted for 24.5% of the variance in students’ grade point averages.

Research has also linked dispositional mindfulness to various positive academic outcomes, including increased working memory capacity (Derakshan and Eysenck, 2009), faster recall (Bakosh et al., 2016; Brown et al., 2016), reduced academic burnout (Aldbyani et al., 2023), and enhanced attentional flexibility (Keye and Pidgeon, 2013; Thompson et al., 2011). Dispositional mindfulness has been found to positively predict academic performance, whereas self-criticism and self-doubt function as negative predictors (Heshmati and Pellerone, 2018). Based on the above-mentioned findings, we propose the following hypothesis:

*H1*: Dispositional mindfulness positively correlates with self-learning and academic achievement.

### Motivation as a mediator

One of the primary concerns for professionals in the field of education is effectively addressing students’ diverse needs. A substantial body of research has examined learner-specific variables—such as personality, motivation, ambition, and learning style—to better understand students’ capabilities and to inform the development of effective educational strategies (Porter and Brophy, 1987). In recent years, there has been growing emphasis on understanding motivation, learning processes, and educational quality. As a result, learner characteristics and motivation have become central concerns in both educational research and practice.

Motivation can arise from intrinsic or extrinsic sources (Ryan and Deci, 2000). It drives goal-directed behavior and sustains individuals’ efforts until their objectives are achieved. According to Stipek (1996), motivation reflects an individual’s capacity to act independently of external social or material reinforcements. Although numerous studies have examined the relationship between mindfulness and motivation, the nature of this relationship remains unclear. For instance, a systematic review by Donald et al. (2020) concluded that mindfulness positively predicts intrinsic motivation while negatively with extrinsic motivation. Another meta-analysis by Li et al. (2023) informed that mindfulness is significantly correlated with intrinsic motivation, but not with extrinsic motivation. This suggests mindfulness may enhance the type of motivation most beneficial for long-term academic and personal success.

Mindfulness theory emphasizes present-moment awareness, attentiveness, and focus, all of which are associated with improved emotional regulation, stress management, and anxiety reduction (Reber, 2014; Tobin, 2018). In contrast, motivation theories—such as self-determination theory—highlight the critical role of motivation in fostering student engagement and predicting academic achievement (Gopalan et al., 2017; Park, 1976; Seifert and Sutton, 2018). According to self-determination theory, mindfulness may influence various forms of motivation, including intrinsic motivation, identified regulation, and external regulation (Ryan et al., 2021).

Given that mindfulness is theorized to influence motivational processes, and motivation is a well-established predictor of both self-learning and academic achievement, it is important to investigate the potential mediating roles of intrinsic and extrinsic motivation. This leads to the following hypothesis:

*H2*: Intrinsic and extrinsic motivations may mediate the association between dispositional mindfulness and both self-learning and academic achievement.

### The current study

Previous research has examined the relationships among dispositional mindfulness, motivation, and academic performance. But the mechanisms linking these variables remain unclear. Motivation has emerged as a potential pathway through which dispositional mindfulness may influence learning outcomes. Self-Determination Theory (van et al., 2012) provides a valuable framework for differentiating between intrinsic motivation, driven by personal interest, and extrinsic motivation, driven by external rewards. While both forms of motivation are associated with academic performance, they may function differently within the context of dispositional mindfulness—an area that remains underexplored, particularly in non-Western and conflict-affected settings.

This study builds upon existing research by testing a dispositional mindfulness→ motivation→ academic performance model within a novel cultural and socioeconomic context: Yemen. The country’s educational systems have experienced chronic disruption due to prolonged conflict, making it a unique and significant setting for this research. The primary aim is to examine whether dispositional mindfulness predicts self-learning and academic achievement among Yemeni college students. A key contribution of this study is its investigation into the distinct mediating roles of intrinsic and extrinsic motivation in this relationship among a conflict-affected population. Therefore, this study seeks to explore whether intrinsic and extrinsic motivation mediate the relationship between dispositional mindfulness, self-learning, and academic achievement in this context. To empirically test these theoretical links, this study uses a cross-sectional design to examine the proposed mediation model in a conflict-affected setting.

## Method

A cross-sectional explanatory study was conducted to examine whether dispositional mindfulness predicts self-learning and academic achievement in Yemeni college students, as well as potential mediating roles of intrinsic and extrinsic motivation in these associations.

### Participants

A total of 283 undergraduate students from Al-Darb Community College in Thamar, Yemen (170 males, 113 females) voluntarily participated in this study, with males representing approximately 60.1% and females 39.9% of the sample. The mean age of participants was 21.3 years (range: 18 to 26 years). An *a priori* power analysis using G*Power 3.1 for a multiple regression with three predictors, an alpha level of 0.05, and an anticipated small-to-medium effect size (Cohen’s *f^2^* = 0.05)—which quantifies the contribution of each predictor to the explained variance—indicated that a minimum of 226 participants was required to achieve 80% power. The final sample (*N* = 283) thus provided sufficient statistical power (~89%). All participants provided informed consent, ensuring the confidentiality and privacy of their responses. Participants did not receive any compensation for their involvement in the study, and data collection was conducted between September and October 2024.

### Instruments

#### Dispositional mindfulness

The Arabic version of the Mindful Attention Awareness Scale (MAAS), which contains 15 items (Baer, 2007), has been proven as a reliable and valid scale in Arabic settings (Aldbyani, 2023). Each item was rated on a 6-point Likert scale, with one indicating “*never or very rarely true*” and six indicating “*very often or always true.*” In this study, Cronbach’s alpha was 0.79. Confirmatory factor analysis indicated acceptable fit (CFI = 0.95, RMSEA = 0.06), The average variance extracted (AVE), which reflects the proportion of variance captured by a construct relative to the variance due to measurement error, exceeded the recommended threshold of 0.50 for all constructs. The composite reliability (CR), which assesses the internal consistency of items within each construct, also surpassed the suggested minimum of 0.70, supporting convergent validity. Additionally, measurement invariance across gender was established at both the configural and metric levels.

#### Self-learning

The Arabic version of the Personal Learning Scale (PLS), which contains 12 items (Lankau and Scandura, 2002), has been proven reliable and valid in Arabic settings. Each item was rated on a 5-point Likert scale—these terms ranged from one (*strongly disagree*) to five (*strongly agree*). In our study, Cronbach’s alpha was 0.81. CFA yielded CFI = 0.96 and RMSEA = 0.05, AVE = 0.55, CR = 0.80, and discriminant validity was confirmed via Fornell–Larcker criteria.

#### Intrinsic and extrinsic motivation

The Arabic version of the Intrinsic and Extrinsic Motivation Scale (IEMS), which contains 43 items (Lepper et al., 2005), has been proven as a reliable and valid scale in Arabic settings (e.g., Muqabalah and AlAdamat, 2021). Each item was rated on a 5-point Likert scale—these terms ranged from one (*strongly disagree*) to five (*strongly agree*). In our study, Cronbach’s alpha was 0.79. Confirmatory analyses supported the two-factor structure (intrinsic vs. extrinsic) with CFI = 0.94 and RMSEA = 0.07; AVE values were 0.50 and 0.51 respectively, and CRs were 0.77 and 0.79, indicating adequate convergent and discriminant validity.

#### Academic achievement

Semester GPA was obtained from official college records and used as an objective indicator of academic performance. At Al-Darb Community College, grades are awarded on a 0–100 scale; GPAs were normalized across disciplines and year levels to ensure comparability.

### Data analysis

Pearson’s correlation coefficients were calculated to examine relationships among dispositional mindfulness, motivation, self-learning, and GPA. Before hypothesis testing, we evaluated regression assumptions: linearity was inspected via scatterplots and residual plots; normality of residuals was confirmed by Shapiro–Wilk tests and Q-Q plots; and multicollinearity was ruled out with variance inflation factors (all VIFs ≤ 2.1). Mediation analyses were conducted in SPSS using the PROCESS macro (version 3.5; Model 4) with 5,000 bootstrap resamples to generate 95% confidence intervals for indirect effects (Hayes, 2012). For each model, we report R^2^ to indicate the proportion of variance explained and compute Cohen’s *f^2^* (*f^2^* = R^2^/(1–R^2^)) to gauge effect size.

## Results

Before starting the final analysis, the entire dataset was thoroughly evaluated for accuracy. No univariate or multivariate outliers or missing values were detected. All regression models met key assumptions. Scatterplots indicated linear relationships; Shapiro–Wilk tests on residuals were non-significant (*p* > 0.10), supporting normality; and VIF values ranged from 1.2 to 2.1, indicating no problematic multicollinearity.

### Correlation among study variables

The results in Table 1 shows that all study variables are significantly and positively correlated (*p* < 0.05). Dispositional mindfulness is moderately associated with self-learning (*r* = 0.493), academic achievement (*r* = 0.428), and both intrinsic (*r* = 0.412) and extrinsic motivation (*r* = 0.392). Self-learning shows a strong correlation with academic achievement (*r* = 0.547), suggesting its key role in academic success. Intrinsic and extrinsic motivation are both positively linked to academic achievement (*r* = 0.490 and *r* = 0.366, respectively), and to each other (*r* = 0.497), indicating that motivational factors are closely related and contribute to academic performance.

**Table 1:** Correlation among study variables.

Variables	Dispositional mindfulness	Self-learning	Academic achievement	Intrinsic motivation	Extrinsic motivation
Dispositional mindfulness	1				
Self-learning	0.493*	1			
Academic achievement	0.428*	0.547*	1		
Intrinsic motivation	0.412*	0.423*	0.490*	1	
Extrinsic motivation	0.392*	0.337*	0.366*	0.497*	1

*p < 0.05. All correlations are Pearson’s r coefficients.

**Figure 1:**
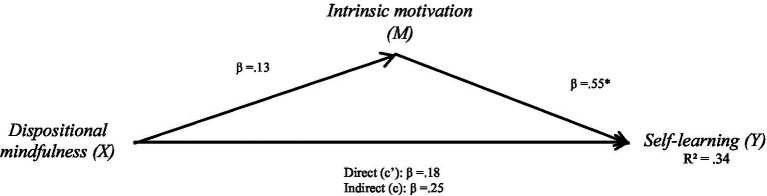
Mediation effect of dispositional mindfulness on self-learning via intrinsic motivation.

Additionally, to account for potential confounding effects, we included gender, age, academic level, and major as control variables in all mediation analyses. None of these control variables had a statistically significant effect on the outcomes (see Figure 1).

#### Mediation effects

This study investigated the influence of dispositional mindfulness on self-learning and academic achievement. The bias-corrected percentile bootstrap method (Model 4 in the SPSS PROCESS macro; sample = 5,000, 95% confidence interval) was used to test the mediating effects. When dispositional mindfulness was included as the independent variable, self-learning and academic achievement as the dependent variables, and intrinsic and extrinsic motivation as the mediating variables, the results were as follows:

#### Self-learning

After controlling for factors that may influence dispositional mindfulness and life satisfaction—such as gender, age, level, and major—the effects of all control variables were found to be non-significant. The total effect of dispositional mindfulness was significant (*β* = 0.25, *p* < 0.05), and the direct effect of dispositional mindfulness on self-learning was also significant (*β* = 0.18, *p* < 0.05), indicating that a one-unit increase in dispositional mindfulness is associated with a 0.18-unit increase in self-learning, after accounting for mediators. Intrinsic and extrinsic motivation were both included as mediators. The bias-corrected percentile bootstrap analysis revealed a significant indirect effect of dispositional mindfulness on self-learning through intrinsic motivation (*ab* = 0.07, *SE* = 0.02, 95% CI [0.03, 0.10]). This suggests a partial mediation, where dispositional mindfulness influences self-learning both directly and indirectly via intrinsic motivation. The total effect (*β* = 0.25) consists of the direct effect (*β* = 0.18) and the indirect effect via intrinsic motivation (*β* = 0.07), indicating that intrinsic motivation accounts for part of the overall relationship. See Table 2 for additional details.

**Table 2:** Mediation effect of dispositional mindfulness on self-learning via intrinsic motivation.

Path	β	SE	95% CI
a. Dispositional Mindfulness→ Intrinsic motivation	0.13	0.02	[0.09, 0.16]
b. Intrinsic motivation→ Self-learning	0.55	0.06	[0.43, 0.66]
c’. Dispositional Mindfulness→ Self-learning (direct effect)	0.18	0.03	[0.12, 0.23]
ab. Indirect via Intrinsic (indirect effect)	0.07	0.02	[0.03, 0.10]
c. Total effect (direct + indirect)	0.25	0.04	[0.17, 0.32]

*p < 0.05.

#### Academic achievement

**Figure 2:**
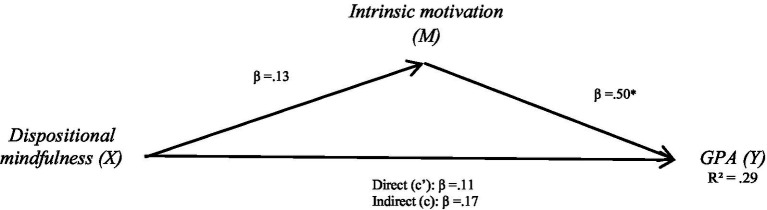
Mediation effect of dispositional mindfulness on academic achievement via intrinsic motivation.

After controlling for factors that may influence dispositional mindfulness and life satisfaction—such as gender, age, level, and major—the effects of all control variables were found to be non-significant. The total effect of dispositional mindfulness was significant (*β* = 0.17, *p* < 0.05), and the direct effect of dispositional mindfulness on academic achievement was also significant (*β* = 0.11, *p* < 0.05), meaning that a one-unit increase in mindfulness is associated with a 0.11-unit increase in academic achievement, independent of the mediators. Both intrinsic and extrinsic motivation were included in the model as mediators. The indirect effect was significant only through intrinsic motivation (*ab* = 0.06, *SE* = 0.02, 95% CI [0.02, 0.09]), while the indirect path through extrinsic motivation was not significant. Although extrinsic motivation was included in the mediation models, its indirect effects on both self-learning (*β* = 0.02, SE = 0.02, 95% CI [−0.01, 0.06]) and academic achievement (*β* = 0.01, SE = 0.02, 95% CI [−0.02, 0.04]) were not statistically significant. This indicates that intrinsic motivation partially mediates the relationship between dispositional mindfulness and academic achievement. Again, the total effect (*β* = 0.17) reflects the sum of the direct effect (*β* = 0.11) and the indirect effect via intrinsic motivation (*β* = 0.06), highlighting the mediating role of intrinsic motivation in linking mindfulness to academic outcomes (see Figure 2).

## Discussion

The first aim of this study was to examine the relationships among dispositional mindfulness, self-learning, and academic achievement. The results indicated that all study variables were positively correlated. Specifically, both intrinsic and extrinsic motivation were positively associated with dispositional mindfulness, self-learning, and academic achievement. These findings underscore the role of dispositional mindfulness in enhancing academic outcomes, aligning with previous research (Bakosh et al., 2016; Heshmati and Pellerone, 2018; Shapiro et al., 2011; Thierry et al., 2016; Zenner et al., 2014). Moreover, the observed positive relationships between mindfulness, academic achievement, and self-learning with both types of motivation highlight the critical role motivation plays in the learning process. This result is consistent with earlier findings (Almalki, 2019; Donald et al., 2020) (see Table 3).

**Table 3:** Mediation effect of dispositional mindfulness on academic achievement via intrinsic motivation.

Model	β	SE	95% CI
a. Dispositional Mindfulness→ Intrinsic motivation	0.13	0.02	[0.09, 0.16]
b. Intrinsic motivation→ GPA	0.50	0.06	[0.38, 0.61]
c’. Dispositional Mindfulness→ GPA (direct effect)	0.11	0.02	[0.07, 0.14]
ab. Indirect via Intrinsic (indirect effect)	0.06	0.02	[0.02, 0.09]
c. Total effect (direct + indirect)	0.17	0.03	[0.11, 0.22]

*p < 0.05, GPA means Academic achievement.

The second aim of this study was to examine the potential mediating roles of intrinsic and extrinsic motivation in the relationship between dispositional mindfulness, self-learning, and academic achievement. The findings revealed that only intrinsic motivation served as a significant mediator in these associations. This result is consistent with previous research, including a meta-analysis by Li et al. (2023) and a systematic review by Donald et al. (2020), both of which reported a significant positive correlation between mindfulness and intrinsic motivation, but no significant association with extrinsic motivation. These findings also support existing theoretical frameworks, including mindfulness theory and motivation theory. Mindfulness theory suggests that a mindful individual is present, attentive, and focused on the current moment, which enhances their ability to regulate emotions, stress, and anxiety (Reber, 2014; Tobin, 2018). In educational settings, a mindful student is more likely to concentrate on the learning process without distraction, thereby fostering intrinsic motivation, increasing engagement, and ultimately contributing to improved academic achievement.

Motivation theories—particularly self-determination theory—propose that motivation, especially intrinsic motivation rooted in personal interest and satisfaction, is a key driver of students’ engagement in learning. According to this theory, intrinsic motivation represents the most enduring and self-determined form of motivation in the educational context, as it is driven by curiosity, enjoyment, and a sense of autonomy (Gopalan et al., 2017; Park, 1976; Seifert and Sutton, 2018). Mindfulness appears to play a significant role in fostering this desire and curiosity. It also enhances self-regulation and discipline, two essential components of academic success, including self-learning and academic achievement. When students are attentive and present, they are better able to independently organize their learning activities and derive satisfaction from the learning process, thereby exemplifying intrinsic motivation. In turn, this intrinsic motivation contributes meaningfully to students’ academic performance.

The absence of a mediating effect of extrinsic motivation may be attributed to the ongoing economic and socio-political challenges in Yemen. More than a decade of conflict, combined with widespread economic hardship, labor market instability, and the collapse of essential infrastructure, has likely diminished the relevance of extrinsic motivators such as academic rewards, grades, or future employment prospects. In such a context, students may be more focused on meeting basic needs and navigating immediate concerns, which can overshadow the influence of external incentives. According to self-determination theory, extrinsic motivation becomes less effective when individuals perceive limited control over external outcomes or when rewards are viewed as uncertain or unattainable. This interpretation aligns with existing research indicating that prolonged conflict and instability can significantly undermine student motivation, particularly extrinsic forms, in conflict-affected settings such as Yemen (Ali, 2019; Garoon, 2022; Taher et al., 2022).

The effects of demographic variables such as gender and age were statistically non-significant. This suggests that the observed associations between dispositional mindfulness, motivation, and academic outcomes are robust across these background characteristics.

Despite the valuable insights offered by this study, several limitations should be acknowledged. First, the use of self-reported measures may introduce biases such as social desirability or inaccurate self-assessment. Second, the cross-sectional research design restricts our ability to draw causal inferences between the variables. Additionally, the study sample consisted exclusively of Yemeni students from a single public college, which limits the generalizability of the findings to other populations or educational contexts. Although we employed a mindfulness scale that has been validated in Arabic settings (Aldbyani, 2023), future research should consider using alternative instruments, such as the Five Facet Mindfulness Questionnaire (FFMQ), to enhance construct validity and facilitate cross-study comparisons.

While our findings support the positive influence of mindfulness on motivation and academic achievement, it is important to recognize that some studies (e.g., Creswell, 2017) have suggested that heightened mindfulness may, in certain contexts, impair performance by disrupting automatic cognitive processes or reducing task efficiency. This possibility warrants further exploration, particularly concerning task complexity and academic setting. Moreover, future research should incorporate multidimensional academic assessments or qualitative evaluations to offer a more comprehensive understanding of students’ academic performance.

Nevertheless, the study successfully highlights the benefits of mindfulness in fostering motivation and enhancing academic achievement. Teachers can encourage students to practice mindfulness regularly by incorporating dispositional mindfulness interventions into their classrooms. Educators should consider structured, evidence-based interventions that integrate mindfulness into the curriculum, for example, brief, guided mindfulness sessions before exams or assignments to help reduce anxiety and enhance focus. At the college level, mindfulness exercises—such as mindful walking, eating, breathing, and socializing—can support students in maintaining strong academic performance. These practices may be particularly effective when tailored to student needs and delivered consistently by trained staff. Mindfulness practices have the potential to improve students’ psychological health and academic outcomes in college settings.

## Data Availability

The datasets presented in this study can be found in online repositories. The names of the repository/repositories and accession number(s) can be found below: The authors confirm that data is available when needed.
